# Roll of Members Medical Association of Georgia, 1885

**Published:** 1886-02

**Authors:** 


### ROLL OF MEMBERS

OF THE

### MEDICAL ASSOCIATION OF GEORGIA,

### 1885.

ARRANGED BY CONGRESSIONAL DISTRICTS.

ABBREVIATIONS—P., President; V-P., Vice President; S., Secretary; C-S., Corresponding Secretary; T., Treasurer; O., Orator; C., Censor—indicating the offices the members hold or have held.

☞ *Members are particularly requested to notify the Secretary promptly on making any change in their post-offices.*

☞ *The Secretary will esteem it a favor if any member will write him at once on noticing any inaccuracy or omission in the roll.*

### FIRST DISTRICT.

| NAME. | POST-OFFICE. | COUNTY. | ADMITTED. |
|---|---|---|---|
| Charlton, T. J. (C.) | Savannah | Chatham | 1862 |
| Colding, C. H. | Savannah | Chatham | 1882 |
| Duncan, Wm. (T.) | Savannah | Chatham | 1868 |
| Elliott, W. H | Savannah | Chatham | 1881 |
| Harris, R. B | Savannah | Chatham | 1885 |
| Houstoun, J. P. S | Savannah | Chatham | 1873 |
| Hummel, George C | Savannah | Chatham | 1884 |
| LeHardy, J. C. (T., V-P., P.) | Savannah | Chatham | 1868 |
| Martin, John D | Savannah | Chatham | 1867 |
| Norton, R. G | Egypt | Effingham | 1872 |
| Nunn, R. J. (C., V-P. P.) | Savannah | Chatham | 1867 |
| Purse, B. S | Savannah | Chatham | 1885 |
| Read, J. B | Savannah | Chatham | 1851 |
| Redding, J. H | Waycross | Ware | 1882 |
| Sample, C. L | Cannoochee | Emanuel | 1875 |
| Sheftall, B. F | Savannah | Chatham | 1878 |
| Stone, George H | Savannah | Chatham | 1875 |

| NAME. | POST-OFFICE. | COUNTY. | ADMITTED. |
|---|---|---|---|
| Quarterman, K. A. | Walthourville | Chatham | 1885 |
| Waring, J. J. | Savannah | Chatham | 1853 |
| Weichelsbaum, J. H. | Savannah | Chatham | 1884 |

### SECOND DISTRICT.

| | | | |
|---|---|---|---|
| Alfriend, E. W. (V-P.) | Albany | Dougherty | 1852 |
| Bruce, W. W | Thomasville | Thomas | 1881 |
| Cheatham, W. B | Dawson | Terrell | 1885 |
| Culpepper, J. T. | Boston | Thomas | 1881 |
| Dekle, Thomas S | Thomasville | Thomas | 1874 |
| Dostor, B. R. (V-P.) | Blakely | Early | 1873 |
| Hillsman, P. L | Albany | Dougherty | 1871 |
| Hopkins, T. S. | Thomasville | Thomas | 1865 |
| McIntosh, T. M. (V-P.) | Thomasville | Thomas | 1874 |
| Strother, Wm. A | Albany | Dougherty | 1873 |
| Talley, H. M | Nashville | Berrien | 1875 |
| Taylor, A. P. | Thomasville | Thomas | 1873 |
| Twitty, W. W. | Camilla | Mitchell | 1871 |
| Wethington, R. T. | Thomasville | Thomas | 1881 |

### THIRD DISTRICT.

| | | | |
|---|---|---|---|
| Bozeman, E. K | Americus | Sumter | 1877 |
| Coleman, J. T. | Perry | Houston | 1851 |
| Fielder, J. F | Eastman | Dodge | 1884 |
| Fort, J. A. | Americus | Sumter | 1883 |
| Havis, M. W | Perry | Houston | 1854 |
| Hawkins, S. B. (V-P.) | Americus | Sumter | 1871 |
| Herrman, J. D | Eastman | Dodge | 1884 |
| Hicks, C. | Dublin | Laurens | 1884 |
| Hightower, R. H | Dublin | Laurens | 1884 |
| Ingram, R. O | Montezuma | Macon | 1884 |
| Jelks, N. P. | Hawkinsville | Pulaski | 1882 |
| Johnson, H. V | Dublin | Laurens | 1874 |
| Joiner, B. L. | Andersonville | Sumter | 1882 |
| Jordan, F. M | Hawkinsville | Pulaski | 1878 |
| Logan, A. J. | Americus | Sumter | 1882 |
| Miller, George T. | Eyronville | Dooly | 1879 |
| Morgan, Y. H | Cochran | Pulaski | 1871 |
| Ross, B. F | Fort Valley | Houston | 1884 |
| Pate, R. H | Snow | Dooly | 1879 |
| Solomon, J. C. | Perry | Houston | 1884 |
| Smith, A. A | Hawkinsville | Pulaski | 1875 |
| Smith, J. B. | Perry | Houston | 1870 |
| Taylor, A. R | Hawkinsville | Pulaski | 1878 |

| NAME. | POST-OFFICE. | COUNTY. | ADMITTED. |
|---|---|---|---|
| Walker, T. F. (v-p.) | Cochran | Pulaski | 1859 |
| Walker, T. D | Cochran | Pulaski | 1882 |

### FOURTH DISTRICT.

| | | | |
|---|---|---|---|
| Bardwell, E. L | Talbotton | Talbot | 1872 |
| Beasley, James A | West Point | Troup | 1884 |
| Bullard, W. L | Columbus | Muscogee | 1882 |
| Calhoun, A. B | Newnan | Coweta | 1874 |
| Cole, J. F | Carrollton | Carroll | 1884 |
| Fitts, W. W | Carrollton | Carroll | 1872 |
| Griggs A. W. (o., v-p., c.) | West Point | Troup | 1866 |
| Griggs, J. W | West Point | Troup | 1878 |
| Grimes, George J | Columbus | Muscogee | 1872 |
| Leitner, C. B | Columbus | Muscogee | 1852 |
| Mitchell, Thomas S | Hamilton | Harris | 1872 |
| Moore, J. T | Whitesburg | Carroll | 1878 |
| Philpot, W. H | Talbotton | Talbot | 1857 |
| Slaughter, J. T | Villa Rica | Carroll | 1859 |
| Smith, C. D | Newnan | Coweta | 1872 |
| Taylor, J. P | Haralson | Coweta | 1859 |
| Wisdom, F. L | Buena Vista | Marion | 1876 |

### FIFTH DISTRICT.

| | | | |
|---|---|---|---|
| Alexander, J. F. (P. C., V-P.) | Atlanta | Fulton | 1849 |
| Anderson, W. A. J | Oxford | Newton | 1873 |
| Armstrong, W. S | Atlanta | Fulton | 1866 |
| Baird, James B. (s. o.) | Atlanta | Fulton | 1872 |
| Bizzell, W. D. (v-p.) | Atlanta | Fulton | 1882 |
| Boring, John M. (v-p.) | Atlanta | Fulton | 1856 |
| Calhoun, A. W. (v-p., c., p.) | Atlanta | Fulton | 1874 |
| Connally, E. L. (c.) | Atlanta | Fulton | 1860 |
| Crawford, G G. (v-p.) | Atlanta | Fulton | 1859 |
| Divine, K. C | Atlanta | Fulton | 1882 |
| Duncan, J. W | Atlanta | Fulton | 1883 |
| Earnest, J G | Atlanta | Fulton | 1882 |
| Elkin, W. S | Atlanta | Fulton | 1882 |
| Evans, W. W | Oxford | Newton | 1872 |
| Faver, Paul | Fayetteville | Fayette | 1873 |
| Gaither, Henry | Oxford | Newton | 1851 |
| Gaston, J. McF | Atlanta | Fulton | 1884 |
| Gray, James A. (s.) | Atlanta | Fulton | 1882 |
| Gwyn, J. P | High Shoals | Walton | 1883 |
| Hamilton, J. L | Stone Mountain | DeKalb | 1859 |
| Hobbs, A. G | Atlanta | Fulton | 1882 |
| Howell, D. H | Atlanta | Fulton | 1878 |

| NAME. | POST-OFFICE. | COUNTY. | ADMITTED. |
|---|---|---|---|
| Jarnagin, W. C | Atlanta | Fulton | 1882 |
| Johnson, J. M | Atlanta | Fulton | 1862 |
| Lallersted, T. L | Panola | DeKalb | 1868 |
| Logan, J. P. (P.) | Atlanta | Fulton | 1858 |
| Love, Wm. Abram (V.-P.) | Atlanta | Fulton | 1866 |
| Miller, H. V. M | Atlanta | Fulton | 1851 |
| Mitchell, T. J | Griffin | Spalding | 1884 |
| Moore, W. C | Clarkston | DeKalb | 1861 |
| Nash, D. T | Lovejoy | Clayton | 1877 |
| Nicolson, W. P | Atlanta | Fulton | 1883 |
| Noble, G. H | Atlanta | Fulton | 1880 |
| O'Brien, F. H | Atlanta | Fulton | 1882 |
| Parks, W. B | Atlanta | Fulton | 1882 |
| Perry, A. C | Covington | Newton | 1878 |
| Pinson, A. J | Lithonia | DeKalb | 1878 |
| Pool, E. W. H | Douglasville | Douglas | 1883 |
| Powell, T. S. (V.-P.) | Atlanta | Fulton | 1857 |
| Quillian, C. C | Atlanta | Fulton | 1884 |
| Roach, E. J | Atlanta | Fulton | 1857 |
| Roach, E. W | Atlanta | Fulton | 1882 |
| Roy, G. G | Atlanta | Fulton | 1878 |
| Scott, H. F. (O.) | Atlanta | Fulton | 1879 |
| Selman, J. L | Douglasville | Douglas | 1882 |
| Stirling, W. L | Atlanta | Fulton | 1878 |
| Taliaferro, V. H. (V.-P.) | Atlanta | Fulton | 1857 |
| Taylor, R. H | Griffin | Spalding | 1879 |
| Todd, J. S. (O., C.) | Atlanta | Fulton | 1872 |
| Westmoreland, W. F. (P.) | Atlanta | Fulton | 1856 |
| Whitley, T. R | Douglasville | Douglas | 1882 |
| Word, R. C | Atlanta | Fulton | 1855 |

### SIXTH DISTRICT.

| | | | |
|---|---|---|---|
| Alexander, L. B. (V-P.) | Forsyth | Monroe | 1877 |
| Barron, R. B | Clinton | Jones | 1884 |
| Blackshear, J. Emmett | Macon | Bibb | 1866 |
| Brown, R. M | Toombsboro | Wilkinson | 1882 |
| Callaway, J. A | Milledgeville | Baldwin | 1882 |
| Cotter, R. O | Macon | Bibb | 1882 |
| Denson, E. J | Bullards | Twiggs | 1884 |
| Duggan, J. H | Stephensville | Wilkinson | 1884 |
| Ferguson, E. G | Macon | Bibb | 1882 |
| Gewinner, N. G | Macon | Bibb | 1884 |
| Gibson, W. C | Macon | Bibb | 1884 |
| Hall, Charles H. (C.) | Macon | Bibb | 1870 |
| Hall, Kenan | Macon | Bibb | 1881 |

| NAME. | POST OFFICE. | COUNTY. | ADMITTED. |
|---|---|---|---|
| Hammond, D. W. (V-P.) | Macon | Bibb | 1857 |
| Harris, I. L | Milledgeville | Baldwin | 1885 |
| Head, J. M | Zebulon | Pike | 1883 |
| Heard, J. H | Walden | Bibb | 1884 |
| Holt, W. F. (V-P., P.) | Macon | Bibb | 1867 |
| Howard, H. L. | Macon | Bibb | 1884 |
| Johnson, J. C | Macon | Bibb | 1875 |
| Jones, L. M | Milledgeville | Baldwin | 1880 |
| Jordan, M. G. W | Culloden | Monroe | 1874 |
| Kenan, Thomas H. (V-P.) | Milledgeville | Baldwin | 1875 |
| King, W | Irwinton | Wilkinson | 1884 |
| Lancaster, J. F | Walden | Bibb | 1884 |
| McHatton, H. | Macon | Bibb | 1883 |
| Moore, K. P. (V-P., O., T., P.) | Macon | Bibb | 1871 |
| O'Daniel, M. H. (O.) | Milledgeville | Baldwin | 188[illegible] |
| O'Daniel, William (T., P.) | Bullards | Twiggs | 1870 |
| Ponder, W. P | Forsyth | Monroe | 188[illegible] |
| Powell, T. O | Milledgeville | Baldwin | 186[illegible] |
| Rosser, W. A | Bolingbroke | Monroe | 188[illegible] |
| Slappey, J. G | Westlake | Twiggs | 187[illegible] |
| Stevens, J. P | Macon | Bibb | 187[illegible] |
| Toole, W. J | Macon | Bibb | 1884 |
| Winchester, W. R | Macon | Bibb | 1884 |
| Whitaker, J. M | Milledgeville | Baldwin | 1881 |
| Williams, H. J (O.) | Macon | Bibb | 1883 |
| Wright, P. H | Macon | Bibb | 1869 |

### SEVENTH DISTRICT.

| | | | |
|---|---|---|---|
| Anderson, R. B | Roswell | Cobb | 1872 |
| Battey, H. H | Rome | Floyd | 1882 |
| Battey, Robert (C., P.) | Rome | Floyd | 1859 |
| Battle, R. J | Cass Station | Bartow | 1879 |
| Bivings, J. C | Dalton | Whitfield | 1873 |
| Calhoun, F. R. (V-P.) | Euharlee | Bartow | 1873 |
| Clements, J. W | Subligna | Chattooga | 1877 |
| Culberson, W. A | Cave Spring | Floyd | 1850 |
| Gordon, C. P | Dalton | Whitfield | 1861 |
| Greene, J. G | Rockmart | Polk | 1879 |
| Holmes, G. W. (V-P., P., C.) | Rome | Floyd | 1867 |
| Holmes, J. B. S | Rome | Floyd | 1872 |
| Holmes, T. M | Rome | Floyd | 1883 |
| Hoyt, W. D | Rome | Floyd | 1871 |
| Humphries, J. R | Acworth | Cobb | 1876 |
| Janes, J. W | Rome | Floyd | 1879 |
| Kendrick, W. S. (O.) | Dirt Town | Chattooga | 1874 |

| NAME | POST-OFFICE. | COUNTY. | ADMITTED. |
|---|---|---|---|
| Quinn. M | Norton | Whitfield | 1883 |
| Richardson, E. H., Jr. (o.c.) | Cedartown | Polk | 1878 |
| Robertson, S | Dallas | Paulding | 1881 |
| Shaw, T. M | Coosa | Floyd | 1878 |
| Wells, W. B. (c., v-p.) | Red Clay | Whitfield | 1873 |
| Wright, A. W | Cave Spring | Floyd | 1879 |

### EIGHTH DISTRICT.

| | | | |
|---|---|---|---|
| Alfriend, F. D | Sparta | Hancock | 1884 |
| Anderson, D. E | Madison | Morgan | 1883 |
| Bell, A. A | Madison | Morgan | 1858 |
| Benedict, Samuel C | Athens | Clarke | 1883 |
| Brawner, W. M | Lexington | Oglethorpe | 1883 |
| Burt, A. Moody | Sparta | Hancock | 1883 |
| Carlton, W. A | Athens | Clarke | 1883 |
| Deadwyler, M. P. (v-p.) | Elberton | Elbert | 1868 |
| Gerdine, John (c.) | Athens | Clarke | 1882 |
| Goss, I. H | Fort Lamar | Madison | 1877 |
| Hale, E. M | Osceola | Oconee | 1883 |
| Hill, J. J | Washington | Wilkes | 1880 |
| Hollingsworth, W. T | Madison | Morgan | 1857 |
| Kennebrew, Henry | Athens | Clarke | 1883 |
| Lane, J. A | Washington | Wilkes | 1880 |
| McClesky, G. L | Athens | Clarke | 1857 |
| Mulligan, G. W | Washington | Wilkes | 1883 |
| Nisbet, R. B | Eatonton | Putnam | 1853 |
| Pope, J. E | Athens | Clarke | 1876 |
| Wade, R. M | Athens | Clarke | 1882 |

### NINTH DISTRICT.

| | | | |
|---|---|---|---|
| Bailey. J. W | Gainesville | Hall | 1861 |
| Hardman, L. G | Harmony Grove | Jackson | 1877 |
| Harris, R. L | Harmony Grove | Jackson | 1883 |
| Neal, W. A | Banksville | Banks | 1883 |
| Pendergrass, J. B | Jefferson | Jackson | 1883 |
| Watson, W. A | Jefferson | Jackson | 1883 |

### TENTH DISTRICT.

| | | | |
|---|---|---|---|
| Allen, J. E | Augusta | Richmond | 1880 |
| Baker, A. H | Augusta | Richmond | 1876 |
| Barnett, J. W | Raytown | Taliaferro | 1880 |
| Battey, W. W | Augusta | Richmond | 1876 |
| Brantley, S. D | Sandersville | Washington | 1849 |

| NAME. | POST-OFFICE. | COUNTY. | ADMITTED. |
|---|---|---|---|
| Campbell, A. Sibley (o., s.) | Augusta | Richmond | 1876 |
| Campbell, H. F. (o. v-p., p.) | Augusta | Richmond | 1851 |
| Carswell, A. W | Herndon | Burke | 1876 |
| Doughty, W. H. (c.) | Augusta | Richmond | 1857 |
| Doughty, W. H., Jr | Augusta | Richmond | 1880 |
| Dugas, George C | Augusta | Richmond | 1880 |
| Flanders, J. W | Wrightsville | Johnson | 1884 |
| Ford DeSaussure (v-p. p) | Augusta | Richmond | 1857 |
| Foster, Eugene (v-p., c., p.) | Augusta | Richmond | 1875 |
| Gay, D. E | Millen | Burke | 1875 |
| Gercke, R O | Augusta | Richmond | 1876 |
| Goodrich, E. C. (t.) | Augusta | Richmond | 1876 |
| Hatton, Joseph | Groveton | Columbia | 1876 |
| Hatch, M. G | Tennille | Washington | 1883 |
| Henderson, J. R | Sun Hill | Washington | 1884 |
| Hickman, C. W | Augusta | Richmond | 1880 |
| Hitt, V. G | Augusta | Richmond | 1870 |
| Holliday, W. Z | Harlem | Columbia | 1884 |
| Hull, J. M | Augusta | Richmond | 1882 |
| Kelley, J. M | Louisville | Jefferson | 1885 |
| Kelly, T. J | Tennille | Washington | 1884 |
| Lane, E. W | Scarboro | Scriven | 1885 |
| Mathis, A. A | Sandersville | Washington | 1872 |
| Roberts, J. B. (v-p.) | Sandersville | Washington | 1875 |
| Smith, Hiram | Augusta | Richmond | 1876 |
| Steiner, H. H | Augusta | Richmond | 1857 |
| Walker, J. L | Wrightsville | Johnson | 1884 |
| Whitehead, A. G. (v-p.) | Waynesboro | Burke | 1869 |
| Wright, J. D | Louisville | Jefferson | 1884 |
| Wright, Thomas R | Augusta | Richmond | 1886 |

### NON-RESIDENTS.

| | | | |
|---|---|---|---|
| Earle, E. P | Birmingham | Alabama | 1882 |
| Haynes, M | | Indian Ter'ry | 1879 |
| Ilyus, E. B | Lancaster | Pennsylvania | 1884 |
| Johnson, J. Thad. (o., s., p.) | Etna | California | 1871 |
| Mitchell, D. L | Cassville | Missouri | 1879 |
| Roach, J. E | Sipes Springs | Texas | 1882 |
| Shi, A. H | Hawthorn | Florida | 1860 |

